# Presentation of retinoblastoma in pregnancy

**DOI:** 10.4103/0301-4738.71682

**Published:** 2010

**Authors:** Mohammad Javed Ali, Santosh G Honavar

**Affiliations:** Ocular Oncology Service, L.V. Prasad Eye Institute, Road No 2, Banjara Hills, Hyderabad - 500 034, India

Dear Editor,

We read with interest the case report by Nandedkar *et al*.[1] We would like to articulate a few of our observations on this.

Fine needle aspiration cytology (FNAC) violates the established protocols in the diagnosis and management of retinoblastoma.[2] Recently, Eide *et al*., have reviewed the entire literature to date and have concluded that FNAC is a relatively strong contraindication in retinoblastoma owing to their poorly cohesive nature.[3] Karcioglu, in a well-controlled study, has histopathologically documented needle tract seeding in cases of retinoblastoma following FNAC.[4] Apart from the dreaded complication of tumor seeding, other possible complications such as vitreous hemorrhage can further preclude tumor assessment.[3]

Advanced retinoblastoma includes International Classification of Intraocular Retinoblastoma Group E, extraocular extension, optic nerve extension, and those with high risk factors for systemic metastasis on histopathology of the enucleated eye.[5] The case reported by the authors fulfills these criteria. There is an established protocol for managing advanced retinoblastoma, which begins with high-dose neoadjuvant chemotherapy aimed to achieve clinical resolution of the optic nerve and/or extraocular component of retinoblastoma as determined by serial computed tomography (CT) scan or magnetic resonance imaging (MRI), followed by extended enucleation, external beam radiotherapy, and adjuvant high-dose chemotherapy for 12 cycles.[5] This protocol helps improve life salvage to over 90% in advanced retinoblastoma. The CT scan provided by the authors does show thickening of the optic nerve. Once there is clinically evident optic nerve or extraocular extension, the case cannot be classified as Group E, rather it then needs to be staged and appropriately managed. Groups A to E are applicable only to intraocular retinoblastoma.[2]

## References

[1] Nandedkar VS, Joshi RA, Kabra N, Deshpande NN (2010). Presentation of retinoblastoma in pregnancy. Indian J Ophthalmol.

[2] Shields JA, Shields CL, Shields JA, Shields CL (2008). Retinoblastoma: Diagnostic approaches. In: Intraocular tumors - an atlas and textbook.

[3] Eide N, Walaas L (2009). Fine needle aspiration biopsy and other biopsies in suspected intraocular malignant disease: A review. Acta Ophthalmol.

[4] Karcioglu ZA, Gordon RA, Karcioglu GL (1985). Tumor seeding in ocular fine needle aspiration biopsy. Ophthalmology.

[5] Honavar SG, Singh AD (2005). Management of advanced retinoblastoma. Ophthalmol Clin North Am.

